# Evolutionary genetics of malaria

**DOI:** 10.3389/fgene.2022.1030463

**Published:** 2022-11-03

**Authors:** Kristan Alexander Schneider, Carola Janette Salas

**Affiliations:** ^1^ Department of Applied Computer- and Biosciences, University of Applied Sciences Mittweida, Mittweida, Germany; ^2^ Department of Parasitology, U.S. Naval Medical Research Unit No 6 (NAMRU-6), Lima, Peru

**Keywords:** complexity of infection (COI), co-infection, mixed-species infection, recrudescence, relapse, seed bank, hypnozoites, multiplicity of infection (MOI)

## Abstract

Many standard-textbook population-genetic results apply to a wide range of species. Sometimes, however, population-genetic models and principles need to be tailored to a particular species. This is particularly true for malaria, which next to tuberculosis and HIV/AIDS ranks among the economically most relevant infectious diseases. Importantly, malaria is not one disease—five human-pathogenic species of *Plasmodium* exist. *P. falciparum* is not only the most severe form of human malaria, but it also causes the majority of infections. The second most relevant species, *P. vivax*, is already considered a neglected disease in several endemic areas. All human-pathogenic species have distinct characteristics that are not only crucial for control and eradication efforts, but also for the population-genetics of the disease. This is particularly true in the context of selection. Namely, fitness is determined by so-called fitness components, which are determined by the parasites live-history, which differs between malaria species. The presence of hypnozoites, i.e., dormant liver-stage parasites, which can cause disease relapses, is a distinct feature of *P. vivax* and *P. ovale* sp. In *P. malariae* inactivated blood-stage parasites can cause a recrudescence years after the infection was clinically cured. To properly describe population-genetic processes, such as the spread of anti-malarial drug resistance, these features must be accounted for appropriately. Here, we introduce and extend a population-genetic framework for the evolutionary dynamics of malaria, which applies to all human-pathogenic malaria species. The model focuses on, but is not limited to, the spread of drug resistance. The framework elucidates how the presence of dormant liver stage or inactivated blood stage parasites that act like seed banks delay evolutionary processes. It is shown that, contrary to standard population-genetic theory, the process of selection and recombination cannot be decoupled in malaria. Furthermore, we discuss the connection between haplotype frequencies, haplotype prevalence, transmission dynamics, and relapses or recrudescence in malaria.

## 1 Introduction

After a decade of declining incidence the number of annual malaria infections rises since 2018, challenging the WHO goal to reduce malaria incidence by at least 90% by 2030 (WHO, 2021a). This is partly attributed to the rapid emergence and spread of anti-malarial drug resistance, an evolutionary-genetic process whose understanding is a global health priority (WHO, 2021b).

Malaria is caused in humans and animals by *Plasmodium* parasites. These unicellular, haploid eukaryotes are transmitted by numerous species of female *Anopheles* mosquitoes. Both the parasite and vector species are adapted to specific human or animal hosts. Five species of *Plasmodium* are pathogenic to humans, which can be transmitted by over 100 *Anopheles* species (Nicoletti, 2020). Over 95% of the 240 million annual infections and 620,000 deaths worldwide are attributed to *P. falciparum*. Although, the WHO recommended the use of RTS,S, the first approved malaria vaccine, in children to prevent *P. falciparum* infections in areas of moderate to high transmission, the vaccine’s efficacy is low and malaria control depends strongly on reliable diagnostics and drug treatments to cure acute infections (Greenwood et al., 2021). While the second most relevant species, *Plasmodium vivax*, receives considerable attention, the other species *P. ovale* sp., *P. malariae*, and *P. knowlesi* are somewhat neglected, due to an outdated distinction between harmful and harmless malaria species (Lover et al., 2018).

The spread of deletions in the histidine-rich protein 2 and 3 (HRP2/3) genes of *P. falciparum*, which encode for the antigens targeted by rapid diagnostic tests (RDTs) as well as drug-resistant *P. falciparum* and *P. vivax* haplotypes substantially challenge successful malaria control. These evolutionary genetic processes are tightly linked to the pathogen’s complex transmission cycle, which besides some species-specific differences, is commonly shared among all *Plasmodia* (Su et al., 2019; Beshir et al., 2022).

The transmission cycle starts with an infected mosquito taking her blood meal. She inoculates parasites in the form of sporozoites from her salivary glands into the human body. This is followed by the exo-erythrocytic cycle, during which sporozoites reach the liver to infect hepatocytes. In the infected liver cells parasites mature into schizonts. The erythrocytic cycle is initiated when the schizonts rupture and merozoites are released into the bloodstream. Erythrocytes are invaded by merozoites, which form ring stage trophozoites and then mature into schizonts. Once they rupture, new merozoites are released into the bloodstream. During this step of asexual reproduction, some parasites differentiate into male or female gametocytes, which do not reproduce in the human host. Once a mosquito ingests male and female gametocytes, the sporogonic cycle is initiated. Gametes released by male and female gametocytes fertilize and form zygotes. Following a step of meiosis, and hence recombination, the zygote becomes tetraploid and develops into ookinetes, which migrate through the midgut wall and transform into oocysts. In the oocyst sporozoite budding occurs in the haploid state. Division of each oocyst produces thousands of sporozoites that move into the mosquito salivary glands, completing the transmission cycle. Because gametocytes immediately release gametes, only parasites exiting the same host recombine, potentially leading to a high degree of inbreeding during the sexual reproduction of the parasite (Ngwa et al., 2016).

Species-specific differences occur in the number of parasites within an infection (parasitemia and gametocytemia counts), and the duration of the various phases in the transmission cycle. The replication of merozoites in 72-hour- rather than 48-hour-cycles distinguishes *P. ovale* sp. from other species. The onset of gametocytogenesis and the longevity of gametocytes were argued to accelerate drug-resistance evolution in *P. falciparum* compared to *P. vivax* (Schneider and Escalante, 2013). Dormant liver-stage parasites (hypnozoites), can result in disease relapses weeks, months, or even years after the clearance of blood stage parasites and occur only in *P. vivax* and *P. ovale* sp. Currently primaquine (PQ) and tafenoquine (TQ) are the only approved drugs to clear hypnozoites (Watson et al., 2021). Unfortunately, patients with (glucose-6 phosphate dehydrogenase) G6PD deficiency, which is widespread in many malaria-endemic areas, cannot be treated with these drugs (Baird et al., 2018; Dean et al., 2020). Extremely prolonged carriage of blood-stage parasites causing recrudescences occur in *P. malariae* (Collins and Jeffery, 2007). It is commonly accepted, although not completely ruled out, that the rebounce of parasitaemia in *P. malariae* is not caused by quiescent pre-erythrocytic stages such as hypnozoites. Because of relapses occurring in *P. vivax*, *P. ovale* sp., and prolonged blood stage parasite carriage in *P. malariae*, these species are resilient in areas in which *P. falciparum* transmission cannot be sustained. While all other human malaria species can—at least in theory—be eradicated by concentrating on the human host, this is not possible for *P. knowlesi*, which is characterized by zoonotic transmission. It became the predominant species in several endemic countries in Southeast Asia, which shifted from malaria control toward elimination (Sutherland, 2016).

The characteristics of the transmission cycle render the application of standard textbook population-genetic results incorrect. Particularly it was shown that the process of selection acting on parasites in the human hosts (including selection for drug resistance) and recombination cannot be separated (Schneider and Kim, 2010). Hence, population-genetic theory and models have to be tailored to the malaria transmission cycle. This has been done mainly for *P. falciparum*. Because a clear path to eradication has been chartered only for *P. falciparum*, the other malaria species gain more importance due to their resilient nature (Lover et al., 2018). This requires to further adapt population-genetic theory to the characteristics of other human-pathogenic malaria species.

Here, we extend a population-genetic framework, originally developed for *P. falciparum*, to be applicable to all other malaria species.

We exemplify the importance of species-specific differences by clarifying the role of hypnozoites in the evolution of drug resistance in *P. vivax* vs. *P. falciparum*. We also clarify, how haplotype frequencies (i.e., their relative abundance in the parasite population) and prevalence (i.e., the likelihood that a given haplotype occurs in an infection) are affected by relapses/recrudescence in other malaria species. Based on this framework, we discuss past and current developments with relevance for the evolutionary genetics of malaria.

## 2 Methods

We extend the population-genetic framework of (Schneider and Kim, 2010; Schneider and Kim, 2011; Schneider, 2021) that describes the temporal change in the distribution of parasite haplotypes due to recombination and selection in generations of transmission cycles. While the original framework was tailored to *P. falciparum*, the extension captures the characteristics of all human-pathogenic malaria species.

**FIGURE 1:**
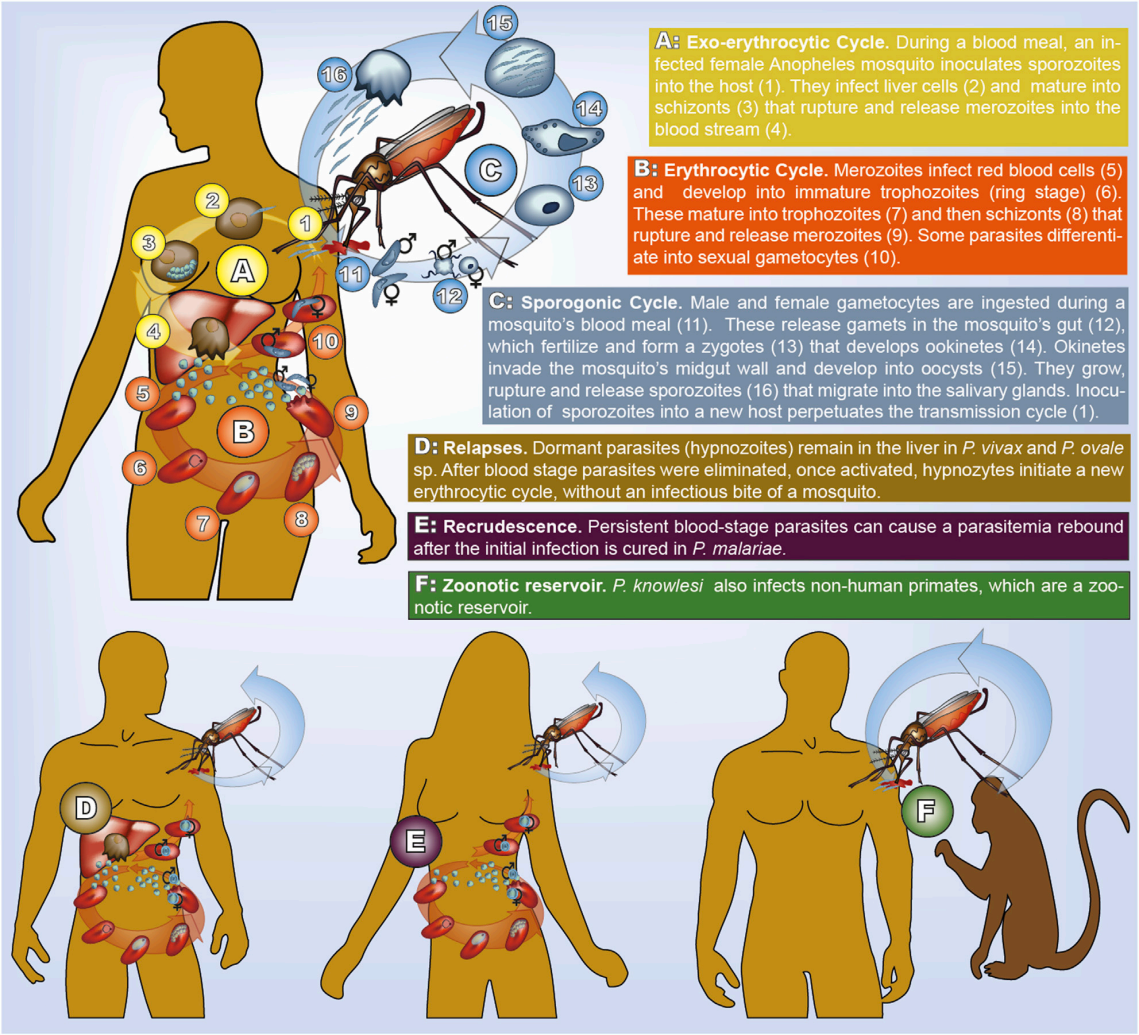
Transmission cycle of human malaria. All species have the same cycle, but parasites life-stages have different morphology (illustrated here for *P. falciparum*). In *P. vivax* and *P. ovale* sp. dormant hypnozoites remain in the liver. In *P. malariae* recrudescence form prolonged blood stage parasites occur. In *P. knowlesi* humans and non-human primates can be infected.

**FIGURE 2:**
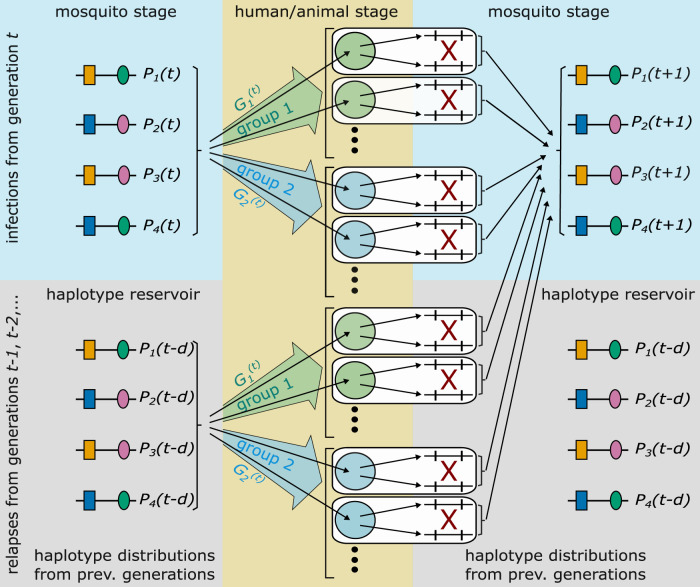
Illustrated is the idealization of the malaria transmission cycle underlying the population-genetic framework. The illustrated genetic architecture of malaria haplotypes assumes two biallelic loci, leading to four possible haplotypes. Furthermore, two groups of hosts are illustrated. Each host is infected by randomly drawing haplotypes from generation *t*, or a relapse/recrudescence from a previous generation occurs, which corresponds to randomly draw parasites from a previous generation (haplotype reservoir). With probability $G_{g}^{\left(t\right)}$ a host belongs to group *g* in generation *t*. The selective environment is different in the two groups. Recombination occurs exclusively between haplotypes exiting the same host. After recombination, haplotypes in the mosquitoes are pooled together to derive their distribution in generation *t* +1.

The model is based on an idealization of the complex malaria transmission cycle (*cf.* Figure 1), which is illustrated in Figure 2. Although, pathogen, mosquito vector, human hosts (and, in the case of *P. knowlesi* the animal host) are involved in transmission, the framework does not require to model transmission dynamics (i.e., the interaction of mosquito vectors and human or animal hosts) explicitly. This conceptional advantages arise, because haplotype frequencies are considered at the end of the sporogenic cycle (*cf.* Figure 2). Thus, the frequency distribution of parasite haplotypes in the mosquitoes’ salivary glands, which are ready for vector-host transmission, is followed.

Host and vector populations are assumed to be sufficiently large and malaria infections sufficiently frequent to justify a deterministic description of the evolutionary dynamics. Steps of full transmission cycles correspond to steps of sexual reproduction, because only one step of sexual reproduction occurs during one full transmission cycle, namely inside the mosquito vector. Many steps of asexual reproduction occur inside the vectors and hosts.

### 2.1 Genetic architecture of haplotypes

The genetic architecture of haplotypes is determined by their allelic configuration at one or several loci. We denote the number of all possible haplotypes by *H*. E.g., *L* biallelic loci lead to *H* = 2^*L*^ haplotypes. In general, if haplotypes are determined by *L* loci, and *n*_*l*_ alleles are segregating at locus *l*, *H* = *n*_1_ ⋅ *n*_2_ ⋅…⋅*n*_*L*_. The frequency of haplotype *h* in generation *t* is denoted by $P_{h}^{\left(t\right)}$ . Collectively, the vector of haplotype frequencies is $P_{t}=\left(P_{1}^{\left(t\right)},\ldots ,P_{H}^{\left(t\right)}\right)$ .

### 2.2 Idealizing the transmission cycle

The idealized transmission cycles allows to describe the evolutionary genetics of malaria in generations of full transmission cycles (Figure 2). In generation *t*, it is assumed that all hosts are infected (or have a relapse or recrudescence) at the same time. Moreover, host-vector transmission is also synchronized. Inside the mosquito, parasites, which were ingested by the mosquitoes, can recombine during one step of sexual reproduction. This determines the distribution of haplotypes in the mosquitoes’ salivary glands of the parasite (sporozoite) population in generation *t* + 1.

#### 2.2.1 Heterogeneity

Disease exposure and transmission intensities are heterogeneous in endemic areas and change over time (e.g. in the context of seasonal transmission) (Bousema et al., 2011; Selvaraj et al., 2018). Moreover, hosts are heterogeneous regarding their level of genetic and naturally acquired immunity, number of co-morbidities, or the drug treatment they receive to cure the infection (in case they receive any), *etc.* (Hedrick, 2011; Gonzales et al., 2020). All of these factors can be addressed by modeling hosts in different groups (strata). Let $G_{g}^{\left(t\right)}$ be the probability that a host, in which an infection occurs in generation *t*, belongs to group *g*. Hence, $G_{1}^{\left(t\right)}+\cdots +G_{S}^{\left(t\right)}=1$ for every generation *t*.

The number of groups, *S*, has to be chosen to capture the features important to the specific application of the framework. For instance, when considering drug resistance evolution, a simple distinction would be between treated and untreated infections, i.e., *S* = 2. In the case of *P. knowlesi* different groups can model human and animal hosts. In the simplest case one would have just two groups (*S* = 2), namely humans and animals.

#### 2.2.2 Relapses and recrudescence

Hosts are not modelled explicitly. This becomes relevant when considering relapses (in *P. vivax* and *P. ovale* sp.) and recrudescence in *P. malariae*. In the following we use relapse and recrudescence synonymously, unless a distinction is necessary.

In the idealized transmission cycle, a relapse in generation *t*, which occurs after a delay of *d* generations, is equivalent to a new infection from the sporozoite population from *d* generations in the past, i.e., from generation *t* − *d*. Let $R_{d}^{\left(t\right)}$ be the probability that an infection in generation *t* is a relapse, with a delay of *d* generations, where $R_{0}^{\left(t\right)}$ is the probability of a new infection at time *t*. Assuming the maximum possible delay is *D*, the relation $\overset{D}{\underset{d=0}{\sum }}R_{d}^{\left(t\right)}=1$ for all *t*, and $1-R_{0}^{\left(t\right)}$ is the probability that a relapse occurs at time *t*.

The framework models the haplotype distribution in generations of transmission cycles not in real-time. The higher the transmission intensities, the more transmission cycles occur per year. The choice of the distribution of relapses has to take this into account (see Results section The effect of recrudescences and relapses). Moreover, the timing of relapses depends on the *Plasmodium* species (White, 2011).

Importantly, a host might have been exposed differently to the disease in the past, i.e., the host might belong to different groups in generations *t* − *d* and *t*. Let $G_{g^{\prime },g}^{\left(t-d,t\right)}$ be the probability that a host, who belonged to group *g*′ in generation *t* − *d*, belongs to group *g* in generation *t* (*d* ≥ 0). Marginalisation yields

$$G_{g}^{\left(t\right)}=\overset{S}{\underset{g^{\prime }=1}{\sum }}G_{g^{\prime },g}^{\left(t-d,t\right)}$$ (1)

for all *t*, *d*, *g*. Hence, the probability that a relapse occurs in generation *t* in a host in group *g* after a delay of *d* generations, when he belonged to group *g*′, is given by

$$R_{d}^{\left(t\right)}G_{g^{\prime },g}^{\left(t-d,t\right)}.$$

### 2.3 Vector-host transmission and multiplicity of infection

**FIGURE 3:**
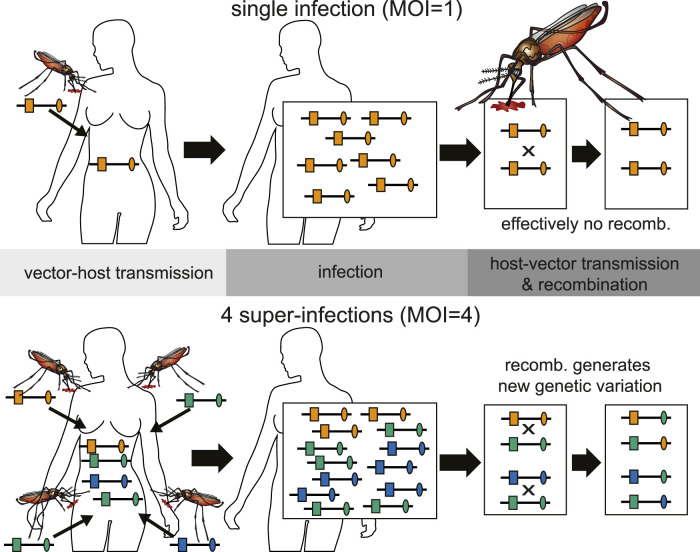
Illustration of the relationship between inbreeding and MOI. Top: An infection with MOI = 1 (single-clone infection) leads only to recombination between clones, i.e., effectively to no recombination. Bottom: Shown is a super-infection with four infective events (MOI = 4) and three different haplotypes being transmitted (one haplotype is transmitted independently by two mosquitoes). Recombination between the illustrated haplotypes leads to the creation of new haplotypes.

The presence of multiple genetically distinct parasite haplotypes within an infection is frequently referred to as multiplicity of infection (MOI) or complexity of infections (COI) and considered important in malaria. The terms MOI and COI are ambiguously defined in the literature (see (Schneider et al., 2022) for a comprehensive review). Although, it is unclear whether MOI is affecting the clinical pathogenesis of malaria, or whether different parasite haplotypes are competing within infections (intra-host competition), MOI mediates the amount of meiotic recombination and scales with transmission intensities (Pacheco et al., 2020) (see Figure 3).

Different parasite haplotypes can occur within an infection, because they are 1) sequentially transmitted (during the course of one disease episode) by different mosquitoes (super-infection); 2) co-transmitted by one mosquito (co-infection); 3) mixed up with parasites from previous infections by relapses or recrudescence.

Concerning models of MOI, the focus was mainly on super-infections. More recently, the importance of co-infections is being emphasized. Namely, more parasite genomics data is being generated, which has enough resolution to study genetic relatedness of parasites. Such data is appropriate for molecular surveillance of transmission routes (Ndiaye et al., 2021). Formal population-genetic frameworks to describe the evolutionary genetics of malaria that consider relapses do not exist. Mathematical models describing relapses in *P. vivax* and *P. ovale* sp. are limited to epidemiological models, e.g., the compartmental model of (Chamchod and Beier, 2013), which neglects parasite genetics. A population-genetic framework applicable to all human-pathogenic malaria species has to be flexible enough to accommodate super-infections, co-infections, relapses, and recrudescence.

To set up the framework an infection is identified by a vector ***m*** = (*m*_1_, … , *m*_*H*_), where *m*_*h*_ is the number of times haplotype *h* is infecting. Hence, *m*_*h*_ = 0 or *m*_*h*_ > 0 if haplotype *h* is absent or present in the infection, respectively. The number *m*_*h*_ accounts for super-infections with the same haplotype. Moreover, it can be interpreted as the “concentration” of haplotype *h* if several haplotypes are co-infecting, *etc.*

Let Pr [***m*** |*t*] be the probability of an infection with configuration ***m*** given generation *t*. The infection might be a new infection or a relapse. The probability of infection ***m***, given it occurs in generation *t*, when the host belongs to group *g*, and given it is a relapse with a delay of *d* generations, when the host belonged to group *g*′, is denoted by Pr [***m*** |*t* − *d*, *g*′; *t*, *g*]. Hence, the probability of infection ***m*** occurring in a host in group *g* in generation *t*, which is a relapse from generation *t* − *d*, when the host belonged to group *g*′, is

$$\mathrm{Pr}\left[m;t-d,g^{\prime };t,g\right]=\mathrm{Pr}\left[m|t-d,g^{\prime };t,g\right]R_{d}^{\left(t\right)}G_{g^{\prime },g}^{\left(t-d,t\right)}.$$ (2)

The conditional probability Pr [***m*** |*t* − *d*, *g*′; *t*, *g*] reflects the model of super- and co-infections. There are many possible models. Super- and co-infections are both notoriously difficult to address. Namely, knowledge about the vector dynamics and the distribution of haplotype combinations in the mosquito population must be known. This is a difficult task and research on the topic is currently expanding, (*cf.* Nkhoma et al., 2012; Wong et al., 2018; Zhu et al., 2019; Nkhoma et al., 2020; Dia and Cheeseman, 2021; Neafsey et al., 2021).

#### 2.3.1 A model for super-infections

Many approaches to estimate MOI or COI by Bayesian or maximum-likelihood methods (e.g. (Hill and Babiker, 1995; Stephens et al., 2001; Rastas et al., 2005; Li et al., 2007; Hastings and Smith, 2008; Druet and Georges, 2010; Ross et al., 2012; Wigger et al., 2013; Taylor et al., 2014; Galinsky et al., 2015; Ken-Dror and Hastings, 2016; Schneider, 2018; Hashemi and Schneider, 2021)) are based on a model, which assumes only super-infections, but no co-infections. The number of super-infections *m* is referred to as multiplicity of infection (MOI; see Figure 3).

Let $M_{m}^{\left(t,g\right)}$ be the probability that a host belonging to group *g* is super-infected exactly *m* times in generation *t*. This is a probability distribution, hence

$$\overset{\infty }{\underset{m=1}{\sum }}M_{m}^{\left(t,g\right)}=1$$ (3)

for all *t* and *g*. At each infectious event, exactly one haplotype is randomly drawn from the mosquito population, i.e., the haplotype distribution ***P***_*t*_. Hence, given MOI *m* in generation *t*, the infection ***m*** = (*m*_1_, … , *m*_*H*_), which indicates how many times haplotype *h* was transmitted, follows a multinomial distribution with parameters *m* and ***P***_*t*_, i.e.,

$$\mathrm{Pr}\left[m|m;t\right]=\left(\begin{array}{c} m\\ m \end{array}\right)P_{t}^{m},$$ (4)

where $(\begin{array}{c} m\\ m \end{array})≔\frac{m!}{\overset{H}{\underset{h=1}{\prod }}m_{h}!}$ is a multinomial coefficient, and $P_{t}^{m}≔\overset{H}{\underset{h=1}{\prod }}{P_{h}^{\left(t\right)}}^{m_{h}}$ . Clearly, the constraint $|m|≔\overset{H}{\underset{h=1}{\sum }}m_{h}=m$ must hold. If an infection is a relapse with a delay of *d* generations, the haplotypes have to be drawn according to the distribution ***P***_*t*−*d*_.

Therefore, the probability of infection ***m*** given it has MOI *m* = | ***m*** | and occurs in generation *t*, when the host belongs to group *g*, from a relapse with a delay of *d* generations, when the host belonged to group *g*′, is given by

$$\mathrm{Pr}\left[m,m|t-d,g^{\prime };t,g\right]=M_{m}^{\left(t-d,g^{\prime }\right)}\left(\begin{array}{c} m\\ m \end{array}\right)P_{t-d}^{m},$$ (5)

where $M_{m}^{\left(t-d,g^{\prime }\right)}$ is the probability of MOI *m* in generation *t* − *d* of a host in group *g*′. This model makes the expression (WHO, 2021b) much more explicit.

#### 2.3.2 Choices for the distribution of super-infections

The model (WHO, 2021b) becomes even more explicit for specific choices of the distribution of MOI. A popular choice emerges from the assumption of rare and independent infections, namely that MOI is conditionally Poisson distributed (*cf.* Schneider, 2021), i.e.,

$$M_{m}^{\left(t,g\right)}=\frac{1}{\exp\left(\lambda_{t,g}\right)-1}\frac{\lambda_{t,g}^{m}}{m!},$$ (6)

where *λ*_*t*,*g*_ > 0 is the Poisson parameter of group *g* in generation *t* and *m* = 1, 2, ….

Another popular choice is the conditional negative-binomial distribution. It is similar to the Poisson distribution but over-dispersed (*cf.* 17).

### 2.4 The exo-erythrocytic and erythrocytic cycles

Assume an infection subsumed by the vector ***m*** having MOI *m* = | ***m*** |. Since all steps of reproduction are clonal inside the host, it is not necessary to model the different parasite stages explicitly. Rather, it suffices to model the change in haplotype frequencies inside the host as a single step.

If the host belongs to group *g*, the ‘absolute’ frequency of haplotype *h* is $\frac{m_{h}}{m}W_{m,h}^{\left(t,g\right)}$ . Here, $W_{m,h}^{\left(t,g\right)}$ is the fitness in generation *t* of haplotype *h* in infection ***m*** of a host belonging to group *g*. It is interpreted as the expected number of gametocyte descendants of a single copy of haplotype *h* infecting the host at the time a mosquito takes her blood meal.

#### 2.4.1 Host-vector transmission

Concerning host-vector transmission, a mosquito ingests a fraction *f* of male and female gametocytes at her blood meal. The gametocyte haplotypes ingested are assumed to be proportional to the haplotype frequencies within the host. More precisely, $f\frac{m_{h}}{m}W_{m,h}^{\left(t,g\right)}$ male and female haplotype *h* are ingested from infection ***m*** in group *g*. (Note different fractions *f* can also be assumed for male and female gametocytes, reflecting an unequal sex ratio.)

#### 2.4.2 Sporogonic cycle

Recombination occurs immediately after the blood meal (see Figure 1), and only parasites descending from the same host can recombine (see Figure 3). Assuming the mosquito bite a host from group *g* with infection ***m***, the probability that a male gamete of haplotype *h* fertilizes a female *i*-gamete is the product of their relative frequencies in the mosquito’s gut, i.e.,

$$\frac{f\frac{m_{h}}{m}W_{m,h}^{\left(t,g\right)}}{fW_{m}^{\left(t,g\right)}}\cdot \frac{f\frac{m_{i}}{m}W_{m,i}^{\left(t,g\right)}}{fW_{m}^{\left(t,g\right)}}=\frac{m_{h}W_{m,h}^{\left(t,g\right)}m_{i}W_{m,i}^{\left(t,g\right)}}{{m^{2}W_{m}^{\left(t,g\right)}}^{2}},$$ (7)

where

$$fW_{m}^{\left(t,g\right)}≔f\overset{H}{\underset{j=1}{\sum }}\frac{m_{j}}{m}W_{m,j}^{\left(t,g\right)}$$ (8)

is the total amount of parasites in the mosquito’s gut. Therefore, the absolute number of such matings is obtained by multiplying the probability of the mating by the total amount of parasites, i.e.,

$$fA_{h,i}^{\left(t,g\right)}$$ (9)

where

$$A_{m,h,i}^{\left(t,g\right)}≔\frac{m_{h}W_{m,h}^{\left(t,g\right)}m_{i}W_{m,i}^{\left(t,g\right)}}{m^{2}W_{m}^{\left(t,g\right)}}.$$ (10)

The absolute frequency of haplotype *h* in the population of mosquitoes, which descends from infections with configuration ***m***, given 1) MOI *m* = | ***m*** |, 2) the infections occur in generation *t*, 3) in hosts in group *g*, which 4) are either novel infections (delay *d* = 0) or relapses with a delay of *d* generations, is

$$\mathrm{Pr}\left[m|m;t-d;t,g\right]\overset{H}{\underset{j,l=1}{\sum }}fA_{m,j,l}^{\left(t,g\right)}r\left(jl\to h\right),$$ (11)

where *r* (*jl* → *h*) is the probability that a mating between gametes with haplotypes *j* and *l* lead to offspring of haplotype *h*.

The absolute number of haplotype *h* in the mosquito population, which descend from hosts in group *g* with MOI *m*, is calculated from the theorem of total probability, i.e., by ‘averaging’ over all possible infections ***m*** with MOI *m*. Incorporating all relapses it is given by

$$P_{h}^{*\left(g,m\right)}\left(t\right)=\overset{D}{\underset{d=0}{\sum }}R_{d}^{\left(t\right)}\underset{m:|m|=m}{\sum }\mathrm{Pr}\left[m|m;t-d;t,g\right]\overset{H}{\underset{j,l=1}{\sum }}fA_{m,j,l}^{\left(t,g\right)}r\left(jl\to h\right).$$ (12)

If an infection in generation *t* is a relapse from generation *t* − *d* the host might have belonged to a different group *g*′ then. Noting, that

$$\mathrm{Pr}\left[m|t-d;t,g\right]=\overset{S}{\underset{g^{\prime }=1}{\sum }}G_{g^{\prime },g}^{\left(t-d,t\right)}\mathrm{Pr}\left[m|m;t-d,g^{\prime };t,g\right]$$ (13)

equation (Su et al., 2019) can be rewritten as

$$P_{h}^{*\left(g,m\right)}\left(t\right)=\overset{D}{\underset{d=0}{\sum }}R_{d}^{\left(t\right)}\overset{S}{\underset{g^{\prime }=1}{\sum }}G_{g^{\prime },g}^{\left(t-d,t\right)}\underset{m:|m|=m}{\sum }\mathrm{Pr}\left[m|m;t-d,g^{\prime };t,g\right]\overset{H}{\underset{j,l=1}{\sum }}fA_{m,j,l}^{\left(t,g\right)}r\left(jl\to h\right).$$ (14)

### 2.5 Evolutionary dynamics

To determine the number of haplotypes *h* in generation *t* + 1, equation (Ngwa et al., 2016) has to be averaged over all possible groups and values of MOI. Hence, the absolute frequency of haplotype *h* in the next generation’s sporozoite population is

$$P_{h}^{*}\left(t+1\right)=f\overset{D}{\underset{d=0}{\sum }}R_{d}^{\left(t\right)}\overset{S}{\underset{g,g^{\prime }=1}{\sum }}G_{g^{\prime },g}^{\left(t-d,t\right)}\overset{\infty }{\underset{m=1}{\sum }}\underset{m:|m|=m}{\sum }\mathrm{Pr}\left[m,m|t-d,g^{\prime };t,g\right]\overset{H}{\underset{j,l=1}{\sum }}A_{m,j,l}^{\left(t,g\right)}r\left(jl\to h\right).$$ (15)

The relative frequency of haplotype *h* in the sporozoite population in generation *t* + 1 is hence

$$P_{h}\left(t+1\right)=\frac{P_{h}^{*}\left(t\right)}{\overset{H}{\underset{i=1}{\sum }}P_{i}^{*}\left(t\right)}.$$ (16)

The dynamics (Watson et al., 2021) are extremely flexible. They allow to model, e.g., temporal changes in selection pressures (for instance changing treatment policies in the context of drug-resistance evolution, temporally varying transmission intensities, intra-host competition of parasites, super- and co-infections, relapses, recrudescences *etc.*). This however requires to specify the model more explicitly.

Next, we show how this is done if only super-infections but no co-infections are considered.

### 2.6 Evolutionary dynamics with super-infections

We introduce a couple of simplifying assumptions, which make the model more explicit. First, only super- but no co-infections are assumed. I.e., the super-infection model (Lover et al., 2018) applies and is substituted into (Schneider and Escalante, 2013). Thus, (Schneider and Escalante, 2013), becomes

Ph∗t+1=f∑d=0DRd(t)∑g,g′=1SGg′,g(t−d,t)∑m=1∞Mm(t−d,g′)∑m:|m|=mmmPt−dm∑j,l=1HAm,j,l(t,g)rjl→h. (17)

## 3 Results

The framework is appropriate to investigate numerous evolutionary-genetics aspects in malaria. It would be far too comprehensive to exemplify the full flexibility. Hence, only special cases are illustrated here. We assume that only super-infections but no co-infections occur, i.e., the dynamics (Baird et al., 2018) are assumed. First, we clarify the difference between haplotype frequency and prevalence. Then we focus on a simple model of drug resistance. Although it is applicable to all malaria species, primarily it shall illustrate the differences between *P. falciparum* and *P. vivax*, because there were no reports on drug resistance in any of the other species (Tseha and Tyagi, 2021).

### 3.1 Frequency and prevalence

The evolutionary genetics of malaria are described as the time-change in the frequency distribution of parasite haplotypes. For instance, monitoring the frequencies of haplotypes, which confer drug resistance is essential. However, concerning the clinical pathogenesis, the occurrence of resistance-conferring haplotypes in infections is more relevant. Due to super- and co-infections the frequency of a haplotype *h*, i.e., its relative abundance among sporozoites in the mosquito population does not coincide with the probability that haplotype *h* occurs in an infection. The latter is referred to as the haplotype’s prevalence.

If only super-infections are considered, the prevalence of haplotype *h* in generation *t*, denoted by $q_{h}^{\left(t\right)}$ is derived in section Prevalence in the Mathematical Appendix. It is given by

$$q_{h}^{\left(t\right)}=1-\overset{D}{\underset{d=0}{\sum }}R_{d}^{\left(t\right)}\overset{S}{\underset{g^{\prime }=1}{\sum }}G_{g^{\prime }}^{\left(t-d\right)}U_{g^{\prime }}^{\left(t-d\right)}\left(1-P_{h}^{\left(t-d\right)}\right),$$ (18)

where $U_{g^{\prime }}^{\left(t-d\right)}\left(x\right)$ is the probability generating function of the MOI distribution in group *g*′ in generation *t* − *d*. This function characterizes transmission in group *g*′ in generation *t* − *d*. From the above expression it is clear that prevalence depends on (i) the frequency of haplotype *h*, (ii) the distributions of MOI in the various groups, and (iii) the distribution of relapses/recrudescence. If no relapses or recrudescences occur, as it is the case for *P. falciparum* and *P. knowlesi*, the prevalence simplifies to

$$q_{h}^{\left(t\right)}=1-\overset{S}{\underset{g=1}{\sum }}G_{g}^{\left(t\right)}U_{g}^{\left(t\right)}\left(1-P_{h}^{\left(t-d\right)}\right).$$ (19)

Hence, for *P. falciparum* and *P. knowlesi* prevalence is characterized by the haplotype frequency distribution in *t*, the distribution of groups, and the MOI distributions in the groups. We illustrate the effect of relapses on prevalence in a simple example below.

### 3.2 Selection at a single locus without intra-host competition

Assume drug resistance is determined by a single locus. This is a reasonable assumption since often drug resistance is determined mainly by mutations at one locus. For instance, in *P. falciparum* resistance to chloroquine is determined by mutations at the *Pfcrt* locus, while resistance artemisinin is determined by mutations in the Kelch-13 propeller region (Cui et al., 2015). The assumption is even justified in sulfadoxine-pyrimethamine resistance, determined by the *Pfdhfr* and *Pfdhps* loci, because mutations at the *Pfdhfr* locus seem to have a much stronger effect (McCollum et al., 2012).

Assume *n* alleles *A*_1_, … , *A*_*n*_ are segregating at the selected locus. The *n* different alleles confer different levels of drug resistance. All other alleles are assumed to be neutral. Thus, the number of possible haplotypes, *H*, is a multiple of *n*, i.e., *H* = *nN*. Hence, *N* is the number of all possible haplotypes when the resistance-conferring locus is disregarded. Let us assume that the haplotypes are ordered such that haplotypes *h* = (*a* − 1)*N* + 1, … , *aN* carry allele *A*_*a*_ at the resistance-conferring locus. Therefore, the frequency of allele *A*_*a*_ at time *t* + 1, denoted by $p_{a}^{\left(t+1\right)}$ is given by

$$p_{a}^{\left(t\right)}=\overset{aN}{\underset{h=\left(a-1\right)N+1}{\sum }}P_{h}^{\left(t\right)}.$$ (20)

Cumulatively, we denote the vector of allele frequencies in generation *t* by ***p***_*t*_.

Under the assumption of no intra-host competition of parasites these dynamics can be made more explicit. In an infection characterized by ***m*** of a host in group *g*, no intra-host competition means that the fitness of an infecting haplotype *h* is independent of what other haplotypes are present in the infection, i.e., it is independent of ***m***, or formally

$$W_{m,h}^{\left(t,g\right)}=W_{h}^{\left(t,g\right)}.$$ (21)

Furthermore, because fitness is only determined by the resistance-conferring locus, the fitness of haplotype *h* depends only on its allele at this locus. Let the fitness of haplotypes carrying allele *A*_*a*_ at the resistance-conferring locus be denoted by $w_{a}^{\left(t,g\right)}$ , i. e,

$$w_{a}^{\left(t,g\right)}=W_{h}^{\left(t,g\right)}=W_{m,h}^{\left(t,g\right)}\;\text{for}\;h=\left(a-1\right)N+1,\ldots ,aN\;\text{and for all}\;m.$$ (22)

Moreover, let the average fitness of allele *A*_*a*_ in generation *t* be

$$w_{a}^{\left(t\right)}=\overset{S}{\underset{g=1}{\sum }}w_{a}^{\left(t,g\right)}G_{g}^{\left(t\right)}.$$ (23)

As shown in the Mathematical Appendix the dynamics of the allele frequencies are given by

$$p_{a}^{\left(t+1\right)}=\frac{w_{a}^{\left(t\right)}\overset{D}{\underset{d=0}{\sum }}R_{d}^{\left(t\right)}p_{a}^{\left(t-d\right)}}{\overset{n}{\underset{b=1}{\sum }}w_{b}^{\left(t\right)}\overset{D}{\underset{d=0}{\sum }}R_{d}^{\left(t\right)}p_{b}^{\left(t-d\right)}}.$$ (24)

As in the case without relapses/recrudescence (*cf.* 17), these dynamics are independent of the distribution of MOI. This holds because no intra-host competition occurs and because only super-infections are considered. Even without intra-host competition the dynamics of the allele frequencies at the selected locus might depend on MOI, depending on the assumed model for co-infections; a general statement cannot be made.

Further, the dynamics (Bousema et al., 2011) depend only on the average fitnesses of the alleles $w_{a}^{\left(t\right)}$ . This implies that the stratification of the host population into different groups does not need to be modelled explicitly, when considering selection at a single locus.

Note that the average fitnesses can be scaled by any constant without affecting the dynamics (Bousema et al., 2011). Hence, it suffices to consider relative fitnesses, and fitness can be normalized such that $w_{1}^{\left(t\right)}=1$ in every generation.

#### 3.2.1 The effect of recrudescences and relapses

In the dynamics of the allele frequencies (Bousema et al., 2011) the effect of relapses or recrudescence is clearly visible. In the case of no relapses or recrudescence, i.e., $R_{d}^{\left(t\right)}=0$ for *d* ≥ 0 the dynamics simplify to

$$p_{a}^{\left(t+1\right)}=\frac{w_{a}^{\left(t\right)}p_{a}^{\left(t\right)}}{\overset{n}{\underset{b=1}{\sum }}w_{b}^{\left(t\right)}p_{b}^{\left(t\right)}}.$$ (25)

In this situation, the allele frequencies in generation *t* + 1 are solely determined by the fitnesses and the allele frequencies in generation *t*. Once relapses or recrudescences are considered, the allele frequencies in generation *t* + 1, depend also on the allele frequencies in previous generations. This is intuitively clear, because relapses/recrudescence are equivalent to infections from the sporozoite population from previous generations (see Figure 2). Hence, relapses/recrudescence act as “seed banks”. Intuitively, this will delay the evolutionary dynamics, because the allele frequencies are averaged over several previous generations.

To further discuss the effect of relapses/recrudescence we impose some additional assumptions. First, we assume that the selective environment does not change over time, i.e., $w_{a}^{\left(t\right)}=w_{a}$ for all *t*. This is a reasonable assumption when considering drug resistance evolution over a time period in which treatment policies do not change. In this case, the change in allele frequencies can be solved explicitly only in the absence of relapses/recrudescence. Namely, the dynamics become

$$p_{a}^{\left(t+1\right)}=\frac{w_{a}^{t+1}p_{a}^{\left(0\right)}}{\overset{n}{\underset{b=1}{\sum }}w_{b}^{t+1}p_{b}^{\left(0\right)}},$$ (26)

where $p_{a}^{\left(0\right)}$ are the initial allele frequencies in generation *t* = 0. From these dynamics it follows that the average fitnesses *w*_*a*_ can be estimated from longitudinal data of allele frequencies by fitting a straight-line regression (see 48, 17).

Once relapses/recrudescence are considered, the dynamics can no longer be solved explicitly, but need to be calculated recursively from the frequencies of the last *D* + 1 generations, i.e., they become

$$p_{a}^{\left(t+1\right)}=\frac{w_{a}\overset{D}{\underset{d=0}{\sum }}R_{d}^{\left(t\right)}p_{a}^{\left(t-d\right)}}{\overset{n}{\underset{b=1}{\sum }}w_{b}\overset{D}{\underset{d=0}{\sum }}R_{d}^{\left(t\right)}p_{b}^{\left(t-d\right)}}.$$ (27)

Importantly, to be able to iterate these dynamics, initial frequencies need to be known from *D* generations in the past. Hence, to calculate the frequencies in generation *t* = 1, initial frequencies $p_{a}^{\left(0\right)},p_{a}^{\left(-1\right)},\ldots ,p_{a}^{\left(-D\right)}$ need to be specified. Moreover, the distribution $R_{d}^{\left(t\right)}$ needs to be known. In practice, the distribution of relapses might change over time. For instance, changes in control policies impact malaria transmission and hence the proportion of new infection in comparison to relapses. If transmission intensities decrease, relapses amount for a larger fraction of infections. Also the number of transmission cycles during 1 year decrease. Because the distribution of the time to relapse measured in years will not change, the time distribution measured in units of transmission cycles will change. In the simplest case the distribution of relapses remains constant over time, i.e., $R_{d}^{\left(t\right)}=R_{d}$ , the change of allele frequencies is given by

$$p_{a}^{\left(t+1\right)}=\frac{w_{a}\overset{D}{\underset{d=0}{\sum }}R_{d}p_{a}^{\left(t-d\right)}}{\overset{n}{\underset{b=1}{\sum }}w_{b}\overset{D}{\underset{d=0}{\sum }}R_{d}p_{b}^{\left(t-d\right)}}.$$ (28)

Unfortunately, even if the distribution of relapses is constant, the average fitnesses can no longer be estimated by a linear regression.

The distribution of relapses depends crucially on the specific parasite strain (White, 2011). Consider the following example of drug-resistance evolution, with just two alleles: allele *A*_1_ being the drug sensitive wildtype and *A*_2_ the mutant allele conferring drug resistance. The mutant allele first occurs in generation *t* = 0 at frequency $p_{2}^{\left(0\right)}=0.001$ . Let *w*_2_ = 1 + *s*, where *s* is the selective advantage of the drug resistant allele *A*_2_. We assume *s* = 0.1, i.e., the fitness is increased by 10%, which is strong selection for population-genetic processes, but reasonable for selection for drug-resistance.

Regarding the distribution of relapses, we assume a situation in which 1 year corresponds to 10 transmission cycles. Relapses often occur in periodic patterns (White, 2011). We first assume a pattern which resembles the relapse pattern described by (Hankey et al., 1953) in temperate zones of Korea. Namely, let *v* be the probability that a malaria episode relapses, i.e., *R*_0_ = 1 − *v*. We assume the first relapse can occur after 10 transmission cycles, and all further relapses after 4 further transmission cycles for a maximum delay of *D* = 90. More precisely, $R_{d}=\frac{v}{21}$ for *d* = 10, 14, 18, 22, … , 90 and *R*_*d*_ = 0 else. As a comparison we assume a simple second pattern of relapses, in which relapses occur 4–50 generations after the initial infection with equal probability, i.e., $R_{d}=\frac{v}{43}$ for *d* = 4, … , 50. Compared with the first pattern, relapses occur more frequently and earlier.

**FIGURE 4:**
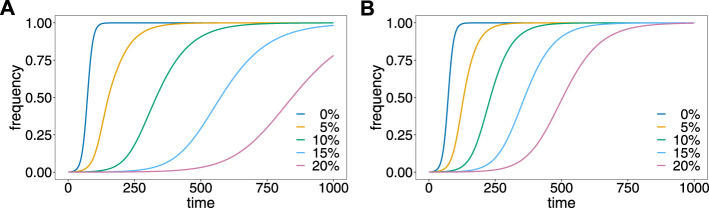
Effect of relapses on the evolutionary dynamics. Shown is the frequency of the resistance-conferring allele as a function of time assuming different proportions, *v* of relapses (colors) for the first **(A)** and second **(B)** patterns of relapses.

The evolutionary dynamics are illustrated in Figure 4. Without relapses *v* = 0, the resistance-conferring allele spreads in approximately 110 generations, which corresponds to 11 years, under the assumed number of 10 transmission cycles per year. Relapses substantially slow down the spread of resistance. The reason is that relapses act like ‘seed banks’ which retain the frequency distribution of previous generations. For the first pattern (Figure 4A), 5% relapses already substantially delay the spread of resistance to about 400 generations or 40 years. With 20% relapses, the frequency of the mutant allele is just 75% after 1,000 generations corresponding to 100 years. For the second pattern (Figure 4B), the results are qualitatively similar, but relapses have a less profound effect, because they occur with shorter delay after the original infection.

These results provide formal evidence that drug resistance spreads faster in *P. falciparum*, where no relapses occur, than in *P. vivax*, where relapses are common. In fact, while drug resistance is a major concern in *P. falciparum*, it is less common in *P. vivax* (Schneider and Escalante, 2013).

The pattern of relapses depends on 1) genetic factors mediating the frequency of their occurrence; 2) transmission intensities determining the number of malaria generations (transmission cycles per year); 3) the fractions of new infections and relapses; and 4) treatment policies. Particularly, if a drug is partnered with primaquine (PQ) or tafenoquine (TQ) for radical cure, the fraction of relapses reduces, accelerating the spread of resistance to the primary treatment. However, since PQ or TQ also act on gametocytes, they prevent transmission and reduce the selective advantage of drug resistance (*cf.* 23).

#### 3.2.2 Prevalence

Next consider the prevalences corresponding to the evolutionary dynamics illustrated in Figure 4. The evolutionary dynamics are determined by the average fitnesses across the groups of hosts and the distribution of relapses. Consequently, it was not necessary to specify the groups explicitly. However, prevalence given by (Collins and Jeffery, 2007) depends on the generating functions of MOI in the different groups. In the simplest case, which we consider here, the whole population consists of only one group (*S* = 1). Furthermore, we assume that the MOI distribution does not change over time, and follows a conditional Poisson distribution (*cf.* Eq. (6)) with parameter *λ*. The generating function of this distribution is given by

$$U\left(x\right)=\frac{\exp\left(\lambda x\right)-1}{\exp\left(\lambda x\right)-1}$$ (29)

(*cf.* 17).

The prevalence of the resistance-conferring allele is obtained from (Collins and Jeffery, 2007) by assuming that haplotypes are characterized by a single locus. Hence,

$$q_{2}^{\left(t\right)}=1-\overset{D}{\underset{d=0}{\sum }}R_{d}U\left(1-p_{2}^{\left(t-d\right)}\right)=\overset{D}{\underset{d=0}{\sum }}R_{d}\frac{1-\exp\left(-\lambda p_{2}^{\left(t-d\right)}\right)}{1-\exp\left(-\lambda \right)}.$$ (30)

**FIGURE 5:**
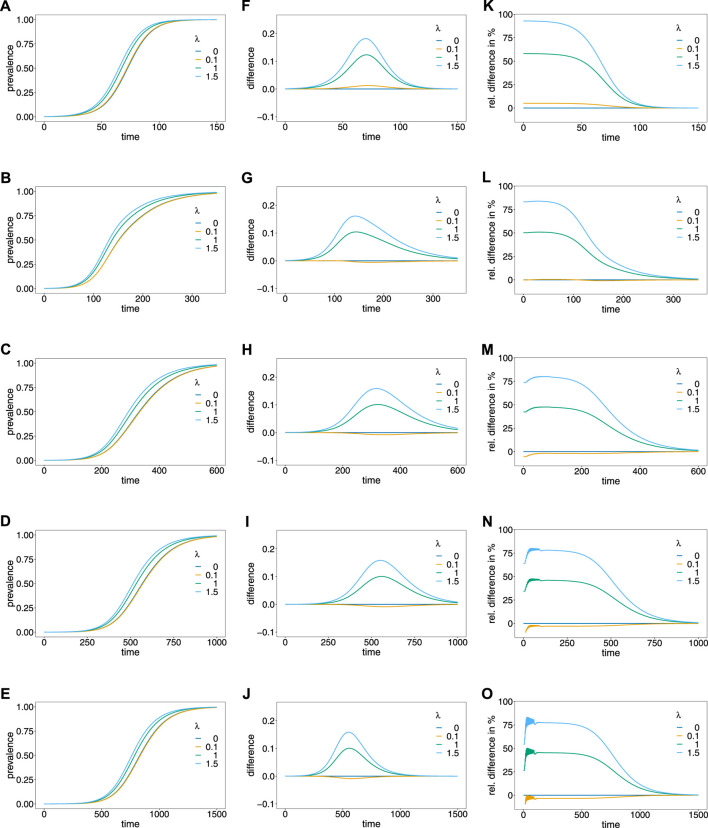
Prevalence. Panels **(A–E)** show the prevalence of the resistance-conferring allele corresponding to the dynamics in Figure 4A for different values of the Poisson parameter *λ* (colors). Panels **(A–E)** correspond to the dynamics with 0%, 5%, 10%, 15%, and 20% relapses, respectively. Panels **(F–J)** show the corresponding difference between prevalence and frequency, and panels **(K–O)** show the corresponding relative difference (prevalence minus frequency divided by frequency) in percent.

The prevalences corresponding to the dynamics illustrated in Figure 4A, are depicted in Figure 5, assuming different values of the Poisson parameter *λ*, corresponding to different transmission intensities.

The case *λ* = 0, implies that only ‘single-infection’ (one infective event) occurs, in which case prevalence and frequency coincide. As shown in (Schneider, 2021) prevalence always exceeds frequency in the case in which no relapses occur (Figures 5A,F,K). This is intuitive, because the likelihood to observe a parasite variant in an infection increases as the average number of super-infections increase. If transmission intensities are intermediate to high (*λ* ≥ 1), prevalence is considerably higher than frequency (Figure 5F). If the frequency of the resistance-conferring allele is small, the difference between frequency and prevalence is small in absolute terms, but high in relative terms (compare Figure 5F with Figure 5K).

If relapses occur, the pattern is similar, however, prevalence can be lower than frequency (see Figures 5F–J). The reason is that prevalence is also determined by the frequency distribution of past generations. This occurs only if the average number of super-infections is small (*λ* slightly larger than 0) and is increasingly pronounced if relapses are more frequent. In general, the difference between frequency and prevalence becomes smaller in absolute and relative terms as the fraction of relapses increase. If this fraction is high (*v* = 0.15 or *v* = 0.2) the particular pattern of relapses leads to oscillations in the relative difference between prevalence and frequency, if the frequency of the resistance-conferring allele is low (see Figures 5N,O).

## 4 Discussion

We introduced a general framework to model evolutionary-genetic processes in malaria, which is flexible enough to capture the characteristics of all human-pathogenic *Plasmodium* species. Such a framework is justified since standard population-genetic theory can only be approximately applied to malaria. The reason is rooted in the malaria transmission cycle, which involves one step of sexual reproduction in the mosquito vectors. A high degree of selfing occurs during this step, because only parasites which descend from the same human host can recombine (*cf.* Figure 3). The framework extends the one introduced in (Schneider and Kim, 2010; Schneider and Kim, 2011; Schneider, 2021), which is only applicable to *P. falciparum*, because it ignores relapses from dormant liver stages as they occur in *P. vivax* and *P. ovale* sp., and recrudescence form prolonged blood stage parasites as they occur in *P. malariae*. These previously widely neglected species are resilient because of relapses and recrudescence, and hence are gaining more importance in the context of malaria eradication. We demonstrated the importance of relapses/recrudescence by contrasting drug resistance-evolution in *P. vivax* and *P. falciparum*.

The necessity to extend the population-genetic framework toward other malaria species is clearly justified by the results presented here. Even in the simplest case of resistance being determined by a single locus, relapses have a profound effect on the evolutionary dynamics, when assuming the same hypothetical drug pressure in both species. Namely, relapses substantially delay the spread of resistance, because they are equivalent—at least in the idealization of the model—to infections with regard to past parasite frequency distributions. In other words, relapses act as seed banks. Dormancy by seed banks is known in evolutionary biology as a bet-hedging strategy that allows organisms to survive through sub-optimal conditions (Shoemaker and Lennon, 2018)—in the case of malaria the absence of the vector. Seed banks are also known to slow down evolutionary processes and influence recombination (Živković and Tellier, 2012; Koopmann et al., 2017; Tellier, 2019). This is no exception in malaria. Although exploring the effect of relapses/recrudescence on recombination was beyond the scope of this work, the effect is rather obvious. Because relapses/recrudescence slow down the evolutionary dynamics, more genetic variation is maintained, leading to a higher level of recombination. In fact, in *P. vivax* higher levels of genetic variations than in *P. falciparum* are a common empirical observation (e.g. (Pacheco et al., 2020)).

Our results have to be understood in a qualitative rather than a quantitative context. Namely, the pattern of relapses have a substantial influence on the evolutionary dynamics. Hence, for adequately predict the spread of resistance, good empirical estimates on the pattern of relapses are necessary. However, empirically distinguishing re-infections (consecutive independent infectious), recrudescence (a rebound of parasitaemia due to incomplete clearance of merozoites), and relapses are notoriously difficult. With more advanced molecular methods becoming available to produce deep-sequencing data (e.g. (Zhong et al., 2018; Gruenberg et al., 2019)), heuristic methods to distinguish recrudescence from reinfections have been proposed (Lin et al., 2015). Also haplotype-based statistical models have been proposed (e.g. (Plucinski et al., 2015)). In principle the framework here can be used to further develop statistical methods to distinguish reinfections from relapses.

To obtain quantitative predictions it is also important to estimate other model parameters. In the context of drug resistance, this includes fitness parameters, metabolic costs for resistance, and the proportion of asymptomatic or untreated infections. The latter can be achieved by routine diagnostics using reliable methods such as ultra-sensitive PCR (e.g. (Gruenberg et al., 2020)). However, also the transmission potential, determined by the abundance of gametocytes has to be determined (*cf.* 9). Selection parameters of drug-resistant haplotypes can be determined from longitudinal molecular data by a linear regressions in *P. falciparum* (McCollum et al., 2012; Schneider, 2021). Disentangling the fitness parameters into metabolic costs and selective advantages of resistance is more difficult. Namely, costs and selective advantages as found in *vitro* studies (*cf.* Cortese and Plowe, 1998) do not linearly scale with *in vivo* observations. In principle, costs can be achieved by contrasting different populations with different drug usage. Comparing such results with *in vitro* studies helps to identify the functional relationship between *in vitro* measurements and *in vivo* observations. Notably, fitness estimates from a linear regression apply mainly to *P. falciparum*. For other malaria species the estimates have to be adapted to the evolutionary dynamics which account for relapses/recrudescence.

Note that the application to modelling drug resistance here had only the purpose of contrasting the absence and presence of relapses. Therefore, only a simplistic model was assumed for drug resistance, i.e., resistance was assumed to be determined by a single biallelic locus. The examples here did not exhibit the full flexibility of the model. If drug resistance occurs in a stepwise fashion as it is found in sulfadoxine-pyrimethamine resistant *P. falciparum* haplotypes (Cortese and Plowe, 1998), where resistance is caused by mutations at several codons in the *Pfdhfr* and *Pfdhps* loci. To capture this situations, resistance-conferring haplotypes have to be modelled by two mulltiallelic loci, where each two-locus haplotype is associated with its own metabolic costs and fitness advantage. Moreover, the mutation haplotypes have to be introduced into the model at different time points. A simple example can be found in (Schneider, 2021).

Relapses are irrelevant in *P. falciparum*, and recrudescences can be neglected, because they occur shortly after the initial infection and do not need to be modeled explicitly. Nevertheless, if transmission intensities are high, which is mainly relevant for *P. falciparum*, the assumption of non-overlapping generations (transmission cycles) are questionable. In the extended framework, relapses can be reinterpreted to mimic overlapping generations. This explains, at least partially, why drug resistance in *P. falciparum* does not necessarily spread first in areas of high transmission (as they occur in Africa) with many more transmission cycles per year.

Reinterpreting relapses in the framework is also important when applied to *P. knowlesi*, which is primarily pathogenic to non-human primates, but became the dominant human-pathogenic malaria species in some endemic areas (Sutherland, 2016). The zoonotic animal-host reservoir renders *P. knowlesi* resilient. Different transmission dynamics between humans and animal hosts can mediate the duration of a transmission cycle. If the number of transmission cycles per year differs among human and non-human primate hosts, this discrepancy can be compensated by modeling overlapping generations by relapses.

We also discussed the differences of frequency and prevalence of parasite haplotypes. The former is the relative abundance of a haplotype in the parasite population, the latter the likelihood that the haplotype occurs in an infection. Studying the haplotype frequency distribution over time is the aim of evolutionary genetics. From a clinical or epidemiological point of view, prevalence is more relevant. The latter is determined by the haplotype frequency distribution and the distribution of super- or co-infections. This was already emphasized in the context of seasonal malaria transmission in (Schneider, 2021) for *P. falciparum*. It was shown that the prevalence of a haplotype always exceeds its frequency. This changes if relapses/recrudescence occur and was exemplified here by the hypothetical dynamics of drug-resistance evolution.

The applications of the framework introduced here are manifold. For instance, in the context of drug resistance, the framework allows to investigate the evolution of multi-drug resistance determined by several loci and changing drug-treatment policies. Also patterns of selection, e.g., genetic hitchhiking, can be studied using this framework. The illustrated applications were only under the simplest assumptions, e.g., of no intra-host competition and super- but no co-infections.

Intra-host competition plays an important role in the spread of HRP2/3 gene deletions associated with false-negative malaria rapid diagnostic tests (RDTs) (Gamboa et al., 2010). Namely, if the treatment guidelines require to verify suspected infections by RDTs before treatment with artemisinin combination therapies (ACTs), as recommended by the WHO (World Health Organization, 2017), false-negative results can lead to delayed treatment. Similarly intra-host competition seems relevant when considering selection on merozoite surface proteins (Goh et al., 2021).

Intra-host dynamics enter the model *via* the definition of fitness. It is not necessary to define an evolutionary-genetic model which captures two timescales, the evolutionary dynamics in terms of generations of transmission cycles, and the timescale of an infectious episode in the same model, as it was done, e.g., in (Kim et al., 2014). Rather, the framework can be used in a multi-scale model, which takes input from a separate intra-host model.

Similarly, the framework does not require to model the mosquito dynamics explicitly. They rather enter *via* the distribution of super- and co-infections. Considering only super-infections has the conceptional advantage, that it is a well-defined model. It is frequently used in statistical approaches to estimate haplotype frequency distributions and MOI (*cf.* e.g. Hill and Babiker, 1995; Stephens et al., 2001; Li et al., 2007; Hastings and Smith, 2008; Wigger et al., 2013; Schneider, 2018; Hashemi and Schneider, 2021). Ignoring co-infections is justified if the distribution of haplotypes in the mosquitoes is uncorrelated or when considering only few loci. However, if one aims to include genetic relatedness, it is important to specify a model for co-infections. This becomes increasingly popular as more high-quality genomic data is becoming available in malaria, which has enough resolution to study genetic relatedness (*cf.* Nkhoma et al., 2012; Wong et al., 2018; Zhu et al., 2019; Nkhoma et al., 2020; Dia and Cheeseman, 2021; Neafsey et al., 2021).

Although the framework is very general, it also has several limitations. First, it ignores mutations. This is not a strong restriction, because in many applications one is interested in *de novo* mutations which occur at discrete time points. This is captured by the model, by introducing new haplotypes (i.e., extending the model) at certain times. However, constant mutation rates, e.g., to study mutation-selection balance, can be easily introduced. Another limitation is the deterministic nature of the framework. When aiming to study stochastic effects such as genetic drift, it is rather straightforward to develop a stochastic version of the framework. Third, the model ignores mitotic recombination during merozoite production inside the host. This plays an important role in some applications, particularly in the structural rearrangement of Var genes (Claessens et al., 2014). These hypervariable genes are responsible to generate important antigen profiles for parasite-host interactions (Warimwe et al., 2009). In any case the framework introduced here allows studying manifold evolutionary-genetic aspects of malaria. Importantly, it allows us to specify benchmark scenarios. More empirical evidence is required to refine relevant parametrizations of the framework.

## Data Availability

The original contributions presented in the study are included in the article/Supplementary Material, further inquiries can be directed to the corresponding author.
