# Supplementary material for: Evolutionary genetics of malaria
Source: Front Genet. 2022 Nov 3;13:1030463. doi: 10.3389/fgene.2022.1030463 (PMC9669584; doi:10.3389/fgene.2022.1030463)
Supplement: Supplementary file 1 [file DataSheet1.pdf]

1 MATHEMATICAL APPENDIX

1.1 Prevalence

The prevalence of haplotype h is the probability that the haplotype occurs in an infection. Such an infection can be a new infection or a relapse for d generations in the past. The general expression is

$$q_h^{(t)} = \sum_{d=0}^{D} R_d^{(t)} \sum_{g,g'=1}^{S} G_{g',g}^{(t-d,t)} \sum_{m=1}^{\infty} \sum_{\substack{\boldsymbol{m}: \\ |\boldsymbol{m}|=m \\ m_h>0}} \Pr[\boldsymbol{m}|m; t-d, g'; t, g],$$

where the innermost sum runs over all possible infections in which haplotype h occurs. Without further specifying the model $\Pr[\boldsymbol{m}|m; t-d, g'; t, g]$, the prevalence cannot be calculated more explicitly.

In the model that assumes only super-infections, the expression for q_h can made more explicit. In this case one obtains

$$q_h^{(t)} = \sum_{d=0}^{D} R_d^{(t)} \sum_{g,g'=1}^{S} G_{g',g}^{(t-d,t)} \sum_{m=1}^{\infty} M_m^{(t-d,g')} \sum_{\substack{\boldsymbol{m}: \\ |\boldsymbol{m}|=m \\ m_h>0}} \binom{m}{\boldsymbol{m}} \boldsymbol{P}_{t-d}^{\boldsymbol{m}}. \tag{A.1}$$

Without loss of generality, we can calculate the prevalence for haplotype H. It is useful to introduce the following notation $\tilde{\boldsymbol{m}} = (m_1, \ldots, m_{H-1})$, $\tilde{\boldsymbol{P}}_t = (P_1^{(t)}, \ldots, P_{H-1}^{(t)})$, $\boldsymbol{m} = (\tilde{\boldsymbol{m}}, m_H)$. First, we simplify the innermost term in (A.1), namely

$$\sum_{\substack{\boldsymbol{m}: \\ |\boldsymbol{m}|=m \\ m_h>0}} \binom{m}{\boldsymbol{m}} \boldsymbol{P}_{t-d}^{\boldsymbol{m}} = \sum_{m_H=1}^{m} \sum_{\substack{\tilde{\boldsymbol{m}}: \\ |\tilde{\boldsymbol{m}}|=m-m_H}} \binom{m}{(\tilde{\boldsymbol{m}}, m_H)} \tilde{\boldsymbol{P}}_{t-d}^{\tilde{\boldsymbol{m}}} P_H^{(t-d)^{m_H}}$$

$$= \sum_{m_H=1}^{m} \binom{m}{m_H} P_H^{(t-d)^{m_H}} \sum_{\substack{\tilde{\boldsymbol{m}}: \\ |\tilde{\boldsymbol{m}}|=m-m_H}} \binom{m-m_H}{\boldsymbol{m}} \tilde{\boldsymbol{P}}_{t-d}^{\tilde{\boldsymbol{m}}}.$$

Application of the multinomial theorem gives

$$\sum_{\substack{\boldsymbol{m}: \\ |\boldsymbol{m}|=m \\ m_h>0}} \binom{m}{\boldsymbol{m}} \boldsymbol{P}_{t-d}^{\boldsymbol{m}} = \sum_{m_H=1}^{m} \binom{m}{m_H} P_H^{(t-d)^{m_H}} \left(\sum_{h=1}^{H-1} P_h^{(t)} \right)^{m_H}.$$

Noting that $1 - P_H^{(t-d)} = \sum_{h=1}^{H-1} P_h^{(t)}$, followed by application of the binomial theorem yields

$$\sum_{\substack{\boldsymbol{m}: \\ |\boldsymbol{m}|=m \\ m_h>0}} \binom{m}{\boldsymbol{m}} \boldsymbol{P}_{t-d}^{\boldsymbol{m}} = \sum_{m_H=1}^{m} \binom{m}{m_H} P_H^{(t-d)^{m_H}} (1 - P_H^{(t-d)})^{m-m_H}$$
$$=1 - \left(1 - P_H^{(t-d)}\right)^m.$$

Substituting this in expression (A.1) yields

$$q_H = \sum_{d=0}^{D} R_d^{(t)} \sum_{g,g'=1}^{S} G_{g',g}^{(t-d,t)} \sum_{m=1}^{\infty} M_m^{(t-d,g')} \left(1 - \left(1 - P_H^{(t-d)}\right)^m\right). \tag{A.2}$$

In the innermost sum one recognizes the probability generating function of the probability distribution $M_m^{(t-d,g')}$, which we denote by $U_{g'}^{(t-d)}$. Therefore,

$$q_H^{(t)} = \sum_{d=0}^{D} R_d^{(t)} \sum_{g,g'=1}^{S} G_{g',g}^{(t-d,t)} \left(1 - U_{g'}^{(t-d)}\left(1 - P_H^{(t-d)}\right)\right)$$
$$=1 - \sum_{d=0}^{D} R_d^{(t)} \sum_{g,g'=1}^{S} G_{g',g}^{(t-d,t)} U_{g'}^{(t-d)}\left(1 - P_H^{(t-d)}\right)$$
$$=1 - \sum_{d=0}^{D} R_d^{(t)} \sum_{g'=1}^{S} U_{g'}^{(t-d)}\left(1 - P_H^{(t-d)}\right) \sum_{g=1}^{S} G_{g',g}^{(t-d,t)}.$$

Similar to (1), marginalization yields

$$G_{g'}^{(t-d)} = \sum_{g=1}^{S} G_{g',g}^{(t-d,t)}.$$

Therefore,

$$q_H^{(t)} = 1 - \sum_{d=0}^{D} R_d^{(t)} \sum_{g'=1}^{S} G_{g'}^{(t-d)} U_{g'}^{(t-d)}\left(1 - P_H^{(t-d)}\right). \tag{A.3}$$

The same calculation holds for each haplotype. Hence, the prevalence of haplotype h in generation t is given by

$$q_h^{(t)} = 1 - \sum_{d=0}^{D} R_d^{(t)} \sum_{g'=1}^{S} G_{g'}^{(t-d)} U_{g'}^{(t-d)}\left(1 - P_h^{(t-d)}\right). \tag{A.4}$$

1.2 Selection at a single locus

Here, we derive the dynamics of allele frequencies if selection occurs only at a single locus. To simplify the notation, we focus on allele A_1 in the following. Using (10) the frequency of A_1 in generation $t+1$ can be expressed as

$$p_1^{(t+1)} = \sum_{h=1}^{N} P_h^{*(t+1)} = \frac{\sum\limits_{h=1}^{N} P_h^{*(t)}}{\sum\limits_{i=1}^{H} P_i^{*(t)}}. \tag{A.5}$$

Since the scaling constant f cancels out in (10), without loss of generality we can set $f = 1$ in the following. Because we assume only super-infections but no co-infections the numerator is given by

$$\sum_{h=1}^{N} P_h^{*(t)} = \sum_{h=1}^{N}\sum_{d=0}^{D} R_d^{(t)} \sum_{g,g'=1}^{S} G_{g',g}^{(t-d,t)} \sum_{m=1}^{\infty} \Bigg[M_m^{(t-d,g')} \times \sum_{\substack{\boldsymbol{m}:\\ |\boldsymbol{m}|=m}} \binom{m}{\boldsymbol{m}} \boldsymbol{P}_{t-d}^{\boldsymbol{m}} \sum_{j,l=1}^{H} A_{\boldsymbol{m},j,l}^{(t,g)} r(jl \to h) \Bigg]. \tag{A.6a}$$

$$= \sum_{d=0}^{D} R_d^{(t)} \sum_{g,g'=1}^{S} G_{g',g}^{(t-d,t)} \sum_{m=1}^{\infty} \Bigg[M_m^{(t-d,g')} \times \sum_{\substack{\boldsymbol{m}:\\ |\boldsymbol{m}|=m}} \binom{m}{\boldsymbol{m}} \boldsymbol{P}_{t-d}^{\boldsymbol{m}} \sum_{j,l=1}^{H} A_{\boldsymbol{m},j,l}^{(t,g)} \sum_{h=1}^{N} r(jl \to h) \Bigg]. \tag{A.6b}$$

For an infection $\boldsymbol{m}$ with MOI m, let c_a be the number of times a haplotype carrying allele A_a was infecting, i.e.,

$$c_a := \sum_{h=(a-1)N+1}^{aN} m_h. \tag{A.7}$$

Clearly, $\sum\limits_{a=1}^{n} c_a = \sum\limits_{h=1}^{H} m_h$. Collectively, we denote the numbers c_a by the vector $\boldsymbol{c} = (c_1, \ldots, c_n) = m$. Note that many different infections can lead to the same vector $\boldsymbol{c}$. If this is the case, we use the notation $\boldsymbol{m} \to \boldsymbol{c}$.

Assume haplotype h carries allele A_a and haplotype i allele A_b at the resistance-conferring locus. The absence of intra-host competition implies

$$A^{(t,g)}_{\boldsymbol{m},h,i} = \frac{m_h w^{(t,g)}_a m_i w^{(t,g)}_b}{m^2 w^{(t,g)}_{\boldsymbol{c}}}, \tag{A.8a}$$

where

$$w^{(t,g)}_{\boldsymbol{c}} := \frac{1}{m}\sum_{a=1}^{n} w^{(t,g)}_a c_a. \tag{A.8b}$$

To simplify (A.6a), we first manipulate the innermost term. We group haplotypes by the alleles they carry at the resistance-conferring locus, i.e,

$$\sum_{j,l=1}^{H} A^{(t,g)}_{\boldsymbol{m},j,l} \sum_{h=1}^{N} r(jl \to h) = \sum_{a=1}^{n}\sum_{b=1}^{n} \sum_{j=(a-1)N+1}^{aN} \sum_{l=(b-1)N+1}^{bN} A^{(t,g)}_{\boldsymbol{m},j,l} \sum_{h=1}^{N} r(jl \to h). \tag{A.9}$$

Since all haplotypes $h = 1, \ldots, N$ carry allele A_1, such a haplotype can only emerge from a mating of haplotypes j and l, if j or l carry allele A_1, i.e., if $j = 1, \ldots, N$ or/and $l = 1, \ldots, N$, otherwise $r(jl \to h) = 0$. Noting first that $A^{(t,g)}_{\boldsymbol{m},j,l} = A^{(t,g)}_{\boldsymbol{m},l,j}$ and $r(jl \to h) = r(jl \to h)$, the above becomes

$$\sum_{j,l=1}^{H} A^{(t,g)}_{\boldsymbol{m},j,l} \sum_{h=1}^{N} r(jl \to h) = \sum_{b=1}^{n} (2 - \delta_{b,1}) \sum_{j=1}^{N} \sum_{l=(b-1)N+1}^{bN} A^{(t,g)}_{\boldsymbol{m},j,l} \sum_{h=1}^{N} r(jl \to h), \tag{A.10}$$

where $\delta_{b,1}$ is the Kronecker delta, i.e., $\delta_{b,1} = 1$ and $\delta_{b,1} = 0$ for $b \neq 1$.

Next consider the innermost expression in (A.10). Assume $b = 1$, i.e., haplotypes j and l carry allele A_1 ($b = 1$ and $2 - \delta_{b,1} = 1$). Hence, any offspring carries carries A_1, i.e., a haplotype $h = 1, \ldots, N$ emerges. Therefore, $\sum_{h=1}^{N} r(jl \to h) = 1$. If only haplotype j carries A_1 but l does not ($b \neq 1$ and $2 - \delta_{b,1} = 2$), only half of the offspring carries allele A_1, i.e., $\sum_{h=1}^{N} r(jl \to h) = \frac{1}{2}$. In any case, $\sum_{h=1}^{N} r(jl \to h)(2 - \delta_{b,1}) = 1$. Therefore, the above simplifies to

$$\sum_{j,l=1}^{H} A^{(t,g)}_{\boldsymbol{m},j,l} \sum_{h=1}^{N} r(jl \to h) = \sum_{b=1}^{n}\sum_{j=1}^{N} \sum_{l=(b-1)N+1}^{bN} A^{(t,g)}_{\boldsymbol{m},j,l}. \tag{A.11}$$

Next assume $\boldsymbol{m} \to \boldsymbol{c}$. Because of (A.8) we can rewrite (A.11) as

$$\sum_{j,l=1}^{H} A_{\boldsymbol{m},j,l}^{(t,g)} \sum_{h=1}^{N} r(jl \to h) = \frac{w_1^{(t,g)}}{m^2 w_{\boldsymbol{c}}^{(t,g)}} \sum_{b=1}^{n} w_b^{(t,g)} \sum_{j=1}^{N} m_j \sum_{l=(b-1)N+1}^{bN} m_l. \tag{A.12}$$

Using first (A.7) and in the next step (A.8b) the above simplifies to

$$\sum_{j,l=1}^{H} A_{\boldsymbol{m},j,l}^{(t,g)} \sum_{h=1}^{N} r(jl \to h) = \frac{w_1^{(t,g)} c_1}{m^2 w_{\boldsymbol{c}}^{(t,g)}} \sum_{b=1}^{n} w_b^{(t,g)} c_b = \frac{w_1^{(t,g)} c_1}{m}. \tag{A.13}$$

Substituting the above into (A.6a) yields

$$\sum_{h=1}^{N} P_h^{*(t)} = \sum_{d=0}^{D} R_d^{(t)} \sum_{g,g'=1}^{S} G_{g',g}^{(t-d,t)} \sum_{m=1}^{\infty} M_m^{(t-d,g')} \sum_{\substack{\boldsymbol{m}: \\ |\boldsymbol{m}|=m}} \binom{m}{\boldsymbol{m}} \boldsymbol{P}_{t-d}^{\boldsymbol{m}} \frac{w_1^{(t,g)} c_1}{m}. \tag{A.14}$$

The innermost term can be rewritten as follows

$$\begin{aligned} \sum_{\substack{\boldsymbol{m}: \\ |\boldsymbol{m}|=m}} \binom{m}{\boldsymbol{m}} \boldsymbol{P}_{t-d}^{\boldsymbol{m}} \frac{w_1^{(t,g)} c_1}{m} &= \sum_{\substack{\boldsymbol{c}: \\ |\boldsymbol{c}|=m}} \sum_{\substack{\boldsymbol{m}: \\ \boldsymbol{m}\to\boldsymbol{c}}} \binom{m}{\boldsymbol{m}} \boldsymbol{P}_{t-d}^{\boldsymbol{m}} \frac{w_1^{(t,g)} c_1}{m} \\ &= \frac{w_1^{(t,g)}}{m} \sum_{\substack{\boldsymbol{c}: \\ |\boldsymbol{c}|=m}} c_1 \sum_{\substack{\boldsymbol{m}: \\ \boldsymbol{m}\to\boldsymbol{c}}} \binom{m}{\boldsymbol{m}} \boldsymbol{P}_{t-d}^{\boldsymbol{m}}. \end{aligned} \tag{A.15}$$

The term in the inner sum is a multinomial expression, which by remembering the definition of allele frequencies (11) can be marginalized to yield

$$\sum_{\substack{\boldsymbol{m}: \\ \boldsymbol{m}\to\boldsymbol{c}}} \binom{m}{\boldsymbol{m}} \boldsymbol{P}_{t-d}^{\boldsymbol{m}} = \binom{m}{\boldsymbol{c}} \boldsymbol{p}_{t-d}^{\boldsymbol{m}}, \tag{A.16}$$

where $\binom{m}{\boldsymbol{c}} = \frac{m!}{\prod_{a=1}^{n} c_a!}$ is a multinomial coefficient, and $\boldsymbol{p}_t^{\boldsymbol{c}} := \prod_{a=1}^{n} (p_a(t))^{c_a}$. Therefore,

$$\sum_{\substack{\boldsymbol{m}: \\ |\boldsymbol{m}|=m}} \binom{m}{\boldsymbol{m}} \boldsymbol{P}_{t-d}^{\boldsymbol{m}} \frac{w_1^{(t,g)} c_1}{m} = \frac{w_1^{(t,g)}}{m} \sum_{\substack{\boldsymbol{c}: \\ |\boldsymbol{c}|=m}} c_1 \binom{m}{\boldsymbol{c}} \boldsymbol{p}_{t-d}^{\boldsymbol{c}}. \tag{A.17}$$

Note that the term in the sum is the expectation of the first component of a multinomially distributed random vector with parameters m and $\boldsymbol{p}_{t-d}$, which is given by $mp_1^{(t-d)}$. This implies

$$\sum_{\substack{\boldsymbol{m}:\\ |\boldsymbol{m}|=m}} \binom{m}{\boldsymbol{m}} \boldsymbol{P}_{t-d}^{\boldsymbol{m}} \frac{w_1^{(t,g)} c_1}{m} = w_1^{(t,g)} p_1^{(t-d)}. \tag{A.18}$$

Combing (A.14) with (A.18) gives

$$\sum_{h=1}^{N} P_h^{*(t)} = \sum_{d=0}^{D} R_d^{(t)} \sum_{g,g'=1}^{S} G_{g',g}^{(t-d,t)} \sum_{m=1}^{\infty} M_m^{(t-d,g')} w_1^{(t,g)} p_1^{(t-d)} \tag{A.19a}$$

$$= \sum_{d=0}^{D} R_d^{(t)} p_1^{(t-d)} \sum_{g,g'=1}^{S} G_{g',g}^{(t-d,t)} w_1^{(t,g)} \sum_{m=1}^{\infty} M_m^{(t-d,g')}. \tag{A.19b}$$

By using (3) and then (1) one obtains

$$\sum_{h=1}^{N} P_h^{*(t)} = \sum_{d=0}^{D} R_d^{(t)} p_1^{(t-d)} \sum_{g,g'=1}^{S} G_{g',g}^{(t-d,t)} w_1^{(t,g)} \tag{A.20a}$$

$$= \sum_{d=0}^{D} R_d^{(t)} p_1^{(t-d)} \sum_{g=1}^{S} w_1^{(t,g)} \sum_{g'=1}^{S} G_{g',g}^{(t-d,t)} \tag{A.20b}$$

$$= \sum_{d=0}^{D} R_d^{(t)} p_1^{(t-d)} \sum_{g=1}^{S} w_1^{(t,g)} G_g^{(t)} \tag{A.20c}$$

$$= w_1^{(t)} \sum_{d=0}^{D} R_d^{(t)} p_1^{(t-d)}. \tag{A.20d}$$

The same calculation can be performed for any allele A_a, i.e.,

$$\sum_{h=(a-1)N+1}^{aN} P_h^{*(t)} = w_a^{(t)} \sum_{d=0}^{D} R_d^{(t)} p_a^{(t-d)}. \tag{A.21}$$

Therefore, the evolutionary dynamics (11) for the change in allele frequencies become

$$p_a^{(t+1)} = \frac{w_a^{(t)} \sum_{d=0}^{D} R_d^{(t)} p_a^{(t-d)}}{\sum_{b=1}^{n} w_b^{(t)} \sum_{d=0}^{D} R_d^{(t)} p_b^{(t-d)}}. \tag{A.22}$$
